# Retraction: PRDM16 upregulation induced by MicroRNA-448 inhibition alleviates atherosclerosis via the TGF-β signaling pathway inactivation

**DOI:** 10.3389/fphys.2023.1327004

**Published:** 2023-10-30

**Authors:** Frontiers Editorial Office

The journal retracts the 11 August 2020 article cited above.

Following publication, concerns were raised regarding the validity of the data in the article. The authors failed to provide the raw data or a satisfactory explanation during the investigation, which was conducted in accordance with Frontiers’ policies. Given the concerns, and the lack of raw data, the editors no longer have confidence in the findings presented in the article.

This retraction was approved by the Chief Editors of Frontiers of Physiology and the Chief Executive Editor of Frontiers. The authors have not responded to correspondence regarding this retraction.

